# HIV-Associated Vacuolar Myelopathy and HIV-Associated Neurocognitive Disorder as an Initial Presentation in HIV Infection

**DOI:** 10.1155/2023/1542785

**Published:** 2023-01-16

**Authors:** Md Asaduzzaman, Atonu Das, Sowmitra Das, Mahfuza Khadija Shamma, Abul Kalam Mohammed Shoab, Biplob Kumar Roy

**Affiliations:** ^1^Department of Medicine, Sylhet MAG Osmani Medical College Hospital, Sylhet-3100, Bangladesh; ^2^Department of Neurology, Sylhet MAG Osmani Medical College Hospital, Sylhet-3100, Bangladesh

## Abstract

Several neurological disorders have been described in HIV infection. Vacuolar myelopathy and neurocognitive disorders usually come at an advanced stage of the disease process. Here, we present a case where these features constitute the presenting complaints. Both of these conditions improved significantly following the start of HAART. We believe this clinical pathway can be a good learning point for the clinician.

## 1. Introduction

Globally, vacuolar myelopathy (VM) is the primary aetiology of myelopathy in HIV/AIDS that is frequently unrecognized during life. VM is usually associated with advanced HIV infection, and it causes the protective myelin sheath of nerve cells in the spinal cord to pull away, creating microscopic holes (vacuoles) in nerve fibers. In the early stage, it is usually asymptomatic. Symptoms become florid when pathology is advanced, which corresponds to the late stage of HIV infection. An autopsy-based case-control study found the presence of VM in 46.5% of autopsies, but surprisingly, signs and symptoms of myelopathy were present only in 26.8% of those with autopsy-proven VM [1]. The typical presentation is slowly progressive and usually painless weakness in the lower extremities, along with walking difficulties, urinary frequency or urgency, and rarely mild sensory disturbances [2].

Neurocognitive disorders in individuals with HIV infection have long been reported. However, the terminology used to name these disorders has changed over time. HAND itself is an umbrella term covering a broad range of neurocognitive deficits. The current gold standard classification scheme for HAND is the Frascati criteria, which provide a set of standards for the detection and assessment of functional declines. Based on the impairment of functional status, this criterion classified HAND into the following three groups: asymptomatic neurocognitive impairment (ANI), mild neurocognitive disorder (MND), and HIV-associated dementia (HAD) [3].

The presentation can range from subtle neuropsychological and motor impairments to profoundly disabling HIV-associated dementia. A recent systematic review and meta-analysis found the prevalence of HAND to be 44.9% among People living with HIV/AIDS (PLWH). However, in the highly active antiretroviral therapy (HAART) era, the incidence of severe forms of neurocognitive disorder has decreased markedly, but milder forms remain [4].

To date, the definitive treatment of VM is still an unresolved issue. The use of HAART has been found to improve symptoms and even imaging features in several reports [5–7], while some other studies observed deterioration following the start of HAART [8]. Previous research revealed that the administration of intravenous immune globulin (IVIG) at high doses can decrease the latent pool of HIV in the dormant memory CD4+ T cells in nine HIV-infected patients [9]. In a trial to assess the effectiveness of IVIg in patients with VM, Cirkurel et al. found lessened lower limb palsy, which may be related to IVIg's anti-inflammatory properties [10]. Based on this evidence, IVIG has been attempted in several other studies, where it is found to be effective in HAART-resistant VM cases [8], but in other studies, it is found to be ineffective [11].

On the other hand, treatment of HAND is more challenging. HAART may improve cognitive function to some extent but not across all domains of neurocognitive function [12, 13]. With a better understanding of the pathophysiology of HAND, numerous adjuvant therapies are also being tested in trials [4, 14].

Herein, we present a case where myelopathy and cognitive disorders were the presenting features of an underlying HIV infection.

## 2. Case Presentation

A 35-year-old female presented to us with complaints of progressive weakness in both lower limbs for 3 months, oral ulcer, and hair loss for 6 months. The weakness developed initially in the right lower extremity and then gradually involved the left lower extremity also. Her weakness progressed over time and did not follow any diurnal variation. Initially, she could walk and do some of her day-to-day work with some level of difficulty. However, the severity of weakness in the lower limbs reached a peak in recent days, which made her unable to do any work, resulting in a completely bedridden state. It is to be mentioned that she has not yet complained of any weakness in the upper limb. She had no history of swallowing or breathing difficulties during this period. She noticed a painless ulcer in her oral cavity, for which she made consultations with several physicians and was prescribed several courses of antibiotics and antifungals without any significant improvement of the lesion. She gave no history of joint pain, tingling/numbness in the extremities, visual impairment, photosensitivity, malar rash (erythematous flat or raised rash across the bridge of the nose and cheeks, which usually spares nasolabial folds), Raynaud's phenomenon (a process in which the fingers and, less commonly, the toes turn different colors (white, blue, and red) in response to certain triggers such as cold or stress and can be associated with discomfort or a “pins and needles” sensation), chronic diarrhea/altered bowel habit, prolonged fever, intake of unpasteurized dairy products, and foreign travel. The patient has been incontinent for urine and stool for the last 1 month. She sometimes could not recognize the feelings of a full bladder or bowel and experienced difficulty making people understand her problems, resulting in urination and defecation in an inappropriate place. She did not have any abnormal urethral or vaginal discharge.

Her attendant reported that she developed cognitive impairment recently. She does not like to talk and wants to avoid the people around her. Sometimes she lost orientation of time and place and developed insidious forgetfulness. The patient did not have any remarkable past medical or surgical history, except the history of cesarean section 12 years ago. During the course of her illness, she noticed progressive weight loss. The patient is married and became a mother of 2 children without any history of fetal loss. Her husband left Bangladesh 2.5 years ago; he was a bus driver by profession at that time. The patient has been amenorrhoeic for the last 6 months. She had never been exposed to extramarital sex before. She had a history of 3 units of whole blood transfusion at the time of her cesarean section. She did not have any history of diabetes mellitus or hypertension. Other systemic inquiry reveals no abnormality.

On examination, the patient was anemic and cachexic (a “wasting” disorder that causes extreme weight loss and muscle wasting). She was apyrexial (absence of fever). The pulse rate was 86 beats/min, blood pressure was 130/70 mmHg without any postural drop. No peripheral lymphadenopathy was palpable. An oral cavity examination revealed the presence of glossitis and oral thrush. Alopecia was also noted. A skin survey revealed normal findings. On neurological system examination, there was hypertonia in both lower limbs with a muscle power of 2/5 on the Medical Research Council (MRC) scale. Muscle wasting was present in both proximal and distal groups of muscle. Deep tendon reflexes in the lower limbs were exaggerated. Knee and patellar clonus were present. Plantar responses were extensor bilaterally. Sensory examinations could not be done properly due to the impairment of the cognitive function of the patient. Examination of the upper limbs was unremarkable. A spinal examination revealed no abnormality. Cerebellar function and cranial nerve were intact. Fundoscopy revealed normal findings. Examination of higher cerebral function unveiled the presence of cognitive dysfunction. It was assessed by the International HIV Dementia Scale (IHDS), which was designed as a screening tool and has been recommended by different HIV guidelines. The IHDS consists of three subtests: a timed finger-tapping test, an alternate hand sequence test, and the recall of four things in two minutes. At first, the patient was instructed to open and close the first two fingers of the nondominant hand as swiftly and broadly as they could for a total of five seconds during the timed finger-tapping subtest. Then, the patient was told to carry out a series of movements with the nondominant hand as quickly as they could over a 10-second period. The third subtest, memory recall, was evaluated by reciting four words to the patient and then asking him to repeat them immediately. Each of these subsets was first demonstrated before the patient was tested. Our patient attained a total score of 2 (timed finger tapping test = 0, alternate hand sequence test = 1, and memory recall = 1). Considering the impaired orientation of the patient, we assessed her functional status by asking questions to her family members. She fulfilled the criteria for HIV-associated dementia (HAND).

**Figure 1:**
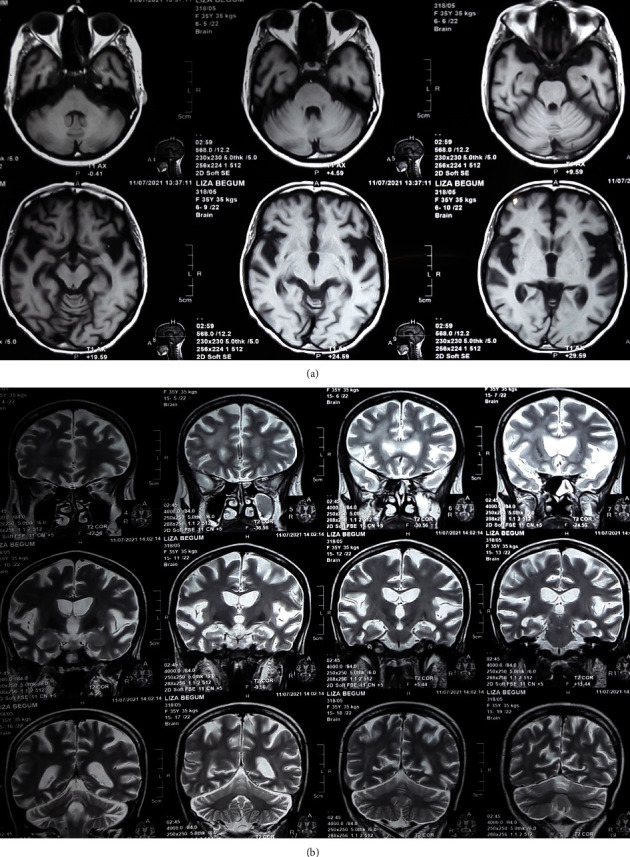
MRI brain: (a) T1 axial view and (b) T2 coronal view, showing generalized brain atrophy.

**Figure 2:**
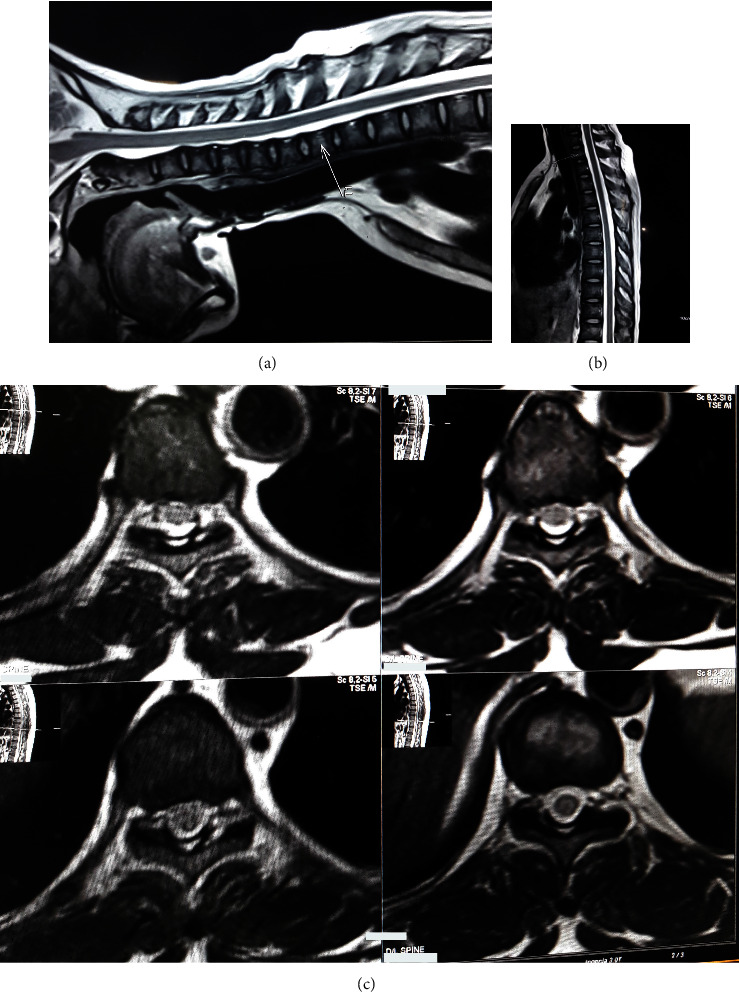
MRI cervical and thoracic spine: (a, b) T2 sagittal and (c) axial view, showing no abnormality.

Initial laboratory investigations (Table 1) revealed the presence of pancytopenia with normocytic normochromic anemia on blood films. Other investigations were notable for the mild elevation of liver enzymes, normal vitamin B12 level (669 pg/ml), normal thyroid function, and negative screens for toxoplasmosis and syphilis. A lumbar puncture showed clear cerebrospinal fluid (CSF). Examination of CSF (Table 2) found pleocytosis and an elevated protein level. Magnetic resonance imaging (MRI) of the brain (Figure 1) showed generalized brain atrophy, which is not age-appropriate. Spinal MRI (Figure 2) revealed no significant lesion. No contrast enhancement was noted anywhere in the cord.

The constellation of progressive paraparesis, neurocognitive dysfunction, bone marrow failure, and weight loss left us with very limited differentials. After any compressive lesion anywhere in the brain or spinal cord, Vit-B12 deficiency and lupus were excluded, the suspicion of HIV infection arrived in our minds. Then we sent for an HIV test, and it came as positive. Her absolute CD4 count was 54/*µ*L. Then, in retrospect, as we revisited her history, we found that all of her physical findings as well as her laboratory report could be explained by HIV-associated VM and HAND. We put the patient on a HAART regimen.

## 3. Follow-Up and Outcomes

At the end of 8 weeks, both clinical features and laboratory parameters (Table 3) were improved. Muscle power became 4/5, which was 2/5 eight weeks ago. Orientation became better. A complete blood count showed improvements in cytopenias. The patient was adherent to the prescribed HAART and has not shown any adverse and unanticipated events so far.

## 4. Discussion

Our case report underlines an unusual presenting feature of HIV infection. Initially, we searched for more common etiologies that could explain her myelopathy and neurocognitive impairment. Following an initial evaluation with no clear-cut diagnosis, we focused on some uncommon etiologies, which prompted us to do an HIV test.

HIV can affect the nervous system in the very early part of the infection. But the virus is isolated rarely from the neurons. HIV seems not to infect neurons directly; rather, it infects macrophages and microglia.

Macrophages probably play a central role in the pathogenesis of neuronal injury in HIV patients [15]. Moreover, the persistence of HIV-1 replication in the macrophage is responsible for continuing inflammation with resultant neuronal injury. Once activated, macrophages, which are an important arm of protection against pathogens, become the effector cells of neuronal damage. Macrophages mediate the damage through several mechanisms. First, interruption of neurotrophin production from microglia. Second, products released from macrophages disrupt astrocyte-neuron interaction, leading to a functional deficit. Thirdly, neurotoxic factors produced by macrophages like platelet-activating factor, nitric oxide, glutamate, arachidonic acid metabolites, and kynurenine metabolites are all involved in the development of neuronal injury and neuron and astrocyte death [16, 17]. Besides, the neurotoxic activities of the viral proteins, especially the viral envelope protein gp120, accessory protein Vpr, and HIV-1 transcriptional activator Tat, also contribute to neuronal damage [18].

Cerebral atrophy, particularly caudate region atrophy, is characteristic of findings in MRI of HIV-associated dementia [19], and evidence suggests that neurological deficits correlate well with brain volume loss [20]. Our patient also had evidence of generalized brain atrophy.

Until now, the most accepted and effective treatment for neurocognitive disorders has been starting HAART. However, for patients who are already on HAART, the treatment strategy is not well defined. Neuropsychological performance, as well as electrophysiologic parameters, shows improvement in HIV/AIDS patients following starting treatment with HAART [21–23]. In our case, we found a significant improvement in clinical status following the start of HAART. Although HAART reduced the prevalence of the severe neurocognitive disorder, it seems to have less effect on milder forms of this disorder. That's why newer therapies are warranted. Considering the role of neuroinflammatory mediators in the pathogenesis of neuronal damage, drugs with different anti-inflammatory and immunomodulatory properties have been investigated in trials with mixed results [24, 25].

In vacuolar myelopathy, the principal target of injury is myelin. Axonal damage occurs secondarily in severe cases. Tumour necrosis factor-*α* and reactive oxygen species produced by macrophages and microglia play a crucial role in myelin damage and vacuole formation [26]. The situation is further complicated by the impairment of the repair mechanism due to a deficiency of S-adenosylmethionine, which results from the adverse effect of macrophage-derived products on the methyl transfer cycle [27].

The disease mainly affects the thoracic spinal cord; less frequently, the cervical and the lumbar cord can be affected. Microscopically, there is the presence of vacuoles in white matter, predominantly in the lateral column, followed by the posterior column. The anterior column can only rarely be affected [28].

As VM is a pathological diagnosis, its onset cannot be identified as premortem. The typical presentation is spastic paraparesis with normal upper extremity function. Our patient had features of a lateral column lesion but no sensory impairment.

Spinal MRI typically shows cord atrophy (predominantly thoracic cord) or signal abnormalities. But imaging findings can be normal in VM [1, 29], which is also depicted in our patient. Before a diagnosis of VM is made, it is essential to exclude the potential differential diagnoses (infectious etiologies, metabolic disorders, lymphoma, structural causes, or neoplastic) based on clinical and imaging findings.

CSF analysis usually reveals pleocytosis and protein elevation [30, 31]. In our study, pleocytosis was present, and the protein level was also increased.

To date, the treatment of vacuolar myelopathy is uncertain; it is mainly supportive, and HAART is believed to improve the prognosis. In addition, there have been case reports of improvement in clinical features and MRI findings of myelopathy following treatment with HAART [8, 32]. We also found a beneficial role of HAART in myelopathy.

## 5. Conclusion

Although the incidence of VM and HAND has decreased a lot in the post-HAART era, this presentation of HIV infection should not be completely forgotten. This case highlights the importance of considering HIV testing in a patient with slowly progressive spastic paraparesis and/or progressive cognitive impairment. We believe this case report would add to the existing knowledge on HIV-VM, HAND, and the role of HAART in treating these conditions and open the door for future research to understand these conditions.

## 6. Limitations

We could not test CSF for HIV viral RNA levels or polymerase chain reaction for other viral studies, including herpes simplex virus (HSV), cytomegalovirus (CMV), Epstein-Barr virus (EBV), varicella-zoster virus (VZV), and JC virus, due to unavailability in our setting.

## Figures and Tables

**Table 1:** Biochemical test.

Laboratory tests	Results	Reference range
Hemoglobin	8.4 gm/dl	12–15.5 g/dL
WBC	4 K/*µ*l	4–10 K/*µ*l
Neutrophil	82%	40–70%
Lymphocyte	12%	20–45%
Platelet count	150 K/*µ*l	150–350 K/*µ*l
PBF	Normocytic normochromic anemia	
ESR	35 mm in 1^st^ hour	0–38
Creatinine	0.8 mg/dl	0.7–1.5 mg/dl
S. electrolyte	Na: 136 mmol/L	Na: 135–145 mmol/L
	K: 3.5 mmol/L	K: 3.5–5 mmol/L
	Cl: 97 mmol/L	Cl: 95–105 mmol/L
	CO_2_: 26 mmol/L	CO_2_: 22–30 mmol/L
S. bilirubin	0.29 mg/dl	0.2–1.2 mg/dl
S. TSH	2.3 mIU/L	0.5–5.0 mIU/L
ALT	131 U/L	Up to 40
Prothrombin time	13 sec	
ANA	13.29 AU/mL	0–40 AU/mL
CPK	22 U/L	30–135
VDRL	Nonreactive	
TPHA	Negative	
Toxoplasma antibodies	Negative	
HBsAg	Negative	
Anti HCV	Negative	
Vitamin B12	669 pg/ml	239–931 pg/ml
HIV Ag/Ab combo assay	Positive	
CD4 count	54/*µ*L	424–1509 *µ*L

PBF, peripheral blood film; ALT, alanine transaminase; ANA, antinuclear antibodies; CPK, creatine phosphokinase; VDRL, venereal disease research laboratory test; TPHA, *Treponema pallidum* hemagglutination assay.

**Table 2:** CSF study.

Name	Results	Reference range
Physical examination	Quantity: 04 ml	
	Color: clear watery	
	Turbidity: absent	
	Clot: absent	
Microscopic examination	Total count: 10/cumm	
	Lymphocyte: 100%	
	Neutrophil: 00%	
	RBC: Nil	
Gram stain	Gram positive cocci: not found	
	Gram negative cocci: not found	
	Gram positive bacilli: not found	
Glucose	49 mg/dl	40–70 mg/dl
Protein	102 mg/dl	15–40 mg/dl
Adenosine deaminase (ADA)	2.0 U/L	0–5 U/L

**Table 3:** Laboratory reports after 8 weeks of receiving HAART.

Laboratory tests	Results	Reference range
Hemoglobin	10.2 g/dl	12–15.5 g/dL
WBC count	6.6 K/*µ*l	4–10 K/*µl*
Platelet count	330 K/*µ*l	150–350 K/*µ*l

## Data Availability

All data generated or analyzed during this study are included in this article.

## Abbreviations

VM: Vacuolar myelopathy
HAND: HIV-associated neurocognitive disorder
HAART: Highly active antiretroviral therapy.
